# Intervertebral disc anomaly intelligent classification system based on deep learning, IDAICS

**DOI:** 10.3389/fradi.2025.1646008

**Published:** 2025-09-09

**Authors:** Zhiheng Gao, Yuchen Qian, Rongkang Fan, Yuqing Yang, Yu Wang, Lei Xing, Yu Chen, Yonggang Li, Haifu Sun, Yusen Qiao

**Affiliations:** ^1^ Department of Orthopaedics, The First Affiliated Hospital of Soochow University, Suzhou, China; ^2^ Department of Otorhinolaryngology, The First Affiliated Hospital of Soochow University, Suzhou, China

**Keywords:** intervertebral disc abnormalities, deep learning, YOLOv8-seg, CT, automated diagnosis, classification accuracy, spinal health

## Abstract

**Background:**

Intervertebral disc anomalies, such as degeneration and herniation, are common causes of spinal disorders, often leading to chronic pain and disability. Accurate diagnosis and classification of these anomalies are critical for determining appropriate treatment strategies. Traditional methods, such as manual image analysis, are prone to subjectivity and time-consuming. With the advancements in deep learning, automated and precise classification of intervertebral disc anomalies has become a promising alternative.

**Objective:**

This study aims to propose a deep learning-based method for classifying intervertebral disc abnormalities, with the goal of improving diagnostic accuracy and clinical efficiency in spinal health management.

**Methods:**

From August 2021 to March 2024, a dataset consisting of 574 CT images of intervertebral discs was collected and labeled into four clinically relevant categories: normal intervertebral discs, Schmorl's nodes, disc bulges, and disc protrusions. The dataset was divided into 500 images for model training, and 74 images for validation. A YOLOv8-seg network was employed for classification, with multiple preprocessing techniques applied to ensure data consistency and enhance model performance.

**Results:**

The IDAICS demonstrated high accuracy in classifying various intervertebral disc anomalies, including disc degeneration, herniation, and bulging, with a classification accuracy of over 93.2%, with a kappa coefficient of 0.905 (*P* < 0.001).

**Conclusion:**

This deep learning-based classification approach provides an efficient and reliable alternative to manual assessment, enabling automated diagnosis of intervertebral disc abnormalities. It offers significant potential to enhance clinical decision-making and improve spinal health management outcomes.

## 1 Introduction

As one of the main manifestations of degenerative spinal disease, disc abnormalities range in condition from the common bulging and herniated discs to the complex Schmorl's nodes. They seriously affect patients' quality of life and can lead to chronic pain, motor dysfunction and even paraplegia (1, 2). In recent years, MRI (Magnetic Resonance Imaging) is a better clinical standard for diagnosing disc abnormalities than CT (Computed Tomography) (3, 4). However, the cost of using MRI is high, the examination time is long, and some patients suffer from claustrophobia and cannot accept MRI examination (5, 6). CT remains widely utilized in clinical practice due to its speed and accessibility, particularly in emergency and outpatient settings, making it an essential diagnostic tool in specific clinical scenarios. And due to the complexity and diversity of clinical imaging data, traditional manual assessment methods are often influenced by subjective factors, resulting in certain limitations in diagnostic accuracy and consistency. Correct diagnosis is a prerequisite for adopting the correct treatment modality and plays a decisive role in the regression of the patient's condition. Therefore, there is an urgent need for a more efficient, accurate, and automated system to assist in the early screening and classification of disc abnormalities.

Manual classification of intervertebral disc abnormalities presents substantial challenges due to subtle morphological differences among conditions. Disc bulges, and protrusions, for instance, often appear visually similar on imaging, though each has distinct characteristics regarding disc shape, size, and displacement. Differentiating these variations requires expertise and is susceptible to influence between observers, even among experienced radiologists. Factors such as image resolution, anatomical variability, and overlapping features across abnormality types further complicate accurate classification. These challenges underscore the need for standardized, automated methods to enhance diagnostic consistency and precision in identifying intervertebral disc conditions. Employing deep learning models for automated classification has the potential to significantly enhance diagnostic accuracy, reduce radiologists' workload, and support more standardized treatment planning, ultimately improving patient outcomes in spinal health care (7–9).

Deep learning has emerged as a vital tool in clinical medicine, especially for reading and diagnosing medical images. By rapidly identifying abnormal structures or regions within patient images, deep learning supports physicians in making accurate diagnostic decisions. Research on deep learning in medical image analysis spans applications including liver (10), pancreas (11), lung (12)and breast cancer (13, 14). Additionally, studies have evaluated AI's potential in tasks such as esophageal segmentation (15) and kidney analysis (16), where reducing inter-observer variability and excluding artifact-affected regions are crucial. Despite these advances, artificial intelligence remains an emerging field in orthopedic imaging, with significant scope for further development (17–21). This study aims to develop an automated classification system for intervertebral disc abnormalities using a novel approach based on the YOLOv8-seg deep learning model, designed to streamline the complex manual diagnosis process. The system categorizes intervertebral disc conditions into four clinically relevant categories: normal intervertebral discs, Schmorl's nodes, disc bulges, and disc protrusions. By achieving accurate classification, the proposed system is expected to support timely clinical decisions, ultimately enhancing patient outcomes.

## 2 Materials and methods

### 2.1 Study subjects

This study utilizes a dataset of CT images, comprising 574 images collected at our hospital from August 2021 to March 2024, representing a range of intervertebral disc conditions. The dataset includes both abnormal cases, such as Schmorl's nodes, disc bulges, and disc protrusions, as well as normal disc images to provide a baseline for comparison. All CT images are 2D slices extracted from 3D scans. Axial and sagittal slices were processed independently, each capturing lesion characteristics from distinct anatomical perspectives, thereby enabling a complementary assessment of disc pathology. To ensure image quality, strict inclusion criteria were applied to exclude images with significant artifacts, low resolution, or incomplete visualization of the disc space. Each image was reviewed and labeled by experienced radiologists, with classifications guided by established clinical and radiological criteria for intervertebral disc conditions. To ensure consistency, the labeling process involved inter-rater agreement among multiple radiologists. Any discrepancies between the radiologists' labels were resolved through consensus discussions, ensuring high agreement and minimizing subjectivity in the final dataset.

### 2.2 YOLOv8-seg-Based intervertebral disc conditions classification

This study proposes a classification method for intervertebral disc conditions using the YOLOv8-seg deep learning network. The output of the model classifies intervertebral disc conditions into one of four categories: normal, Schmorl's nodes, bulge, or protrusion. Both axial and sagittal view slices were utilized in the model to provide comprehensive information. The YOLOv8-seg model is trained on a large dataset of pre-labeled CT images, each depicting distinct disc conditions. By extracting unique features for each type of intervertebral disc condition, the model uses these features as inputs to classify corresponding test images with high accuracy. This approach enables the model to reliably distinguish between normal intervertebral discs, Schmorl's nodes, disc bulges, and disc protrusions, thereby supporting automated and consistent diagnosis. The model was trained on an Nvidia 4090 24GB GPU, providing the computational power necessary for efficient model training and high-performance processing of large image datasets.

#### 2.2.1 Data preprocessing

To optimize the CT images for input into the YOLOv8-seg model, several preprocessing steps were applied. First, the images were standardized by converting them from their original black-and-white format to grayscale. This conversion reduces the number of image channels, which enhances processing efficiency. It is especially important because the global training label is a grayscale image with a single channel, where pixel values range from 0 to 255. In this system, pixel values corresponding to the four intervertebral disc types are labeled as 1, 2, 3, and 4, respectively, while the background pixels are assigned a value of 0. Grayscale conversion was carried out using the Python PIL library, ensuring consistency between the input images and training labels. In addition, significant variations in image scale can lead to inconsistencies in feature extraction, adversely affecting the speed and accuracy of model training. To address this issue and mitigate the impact of scale variations on feature extraction, the images were resized to a uniform resolution(512 × 512 pixels), ensuring they met the input requirements of the YOLOv8-seg model. This resizing process was also performed using the PIL library, standardizing the image dimensions for the iterative learning network. To alleviate the imbalance of dataset samples, we applied real-time data augmentation techniques during the training phase, including horizontal flipping. These enhancements were implemented through the built-in augmentation module of YOLOv8-seg, aiming to improve the generalization ability of the model without introducing bias into the test set.

#### 2.2.2 Dataset construction

**Figure 1:**
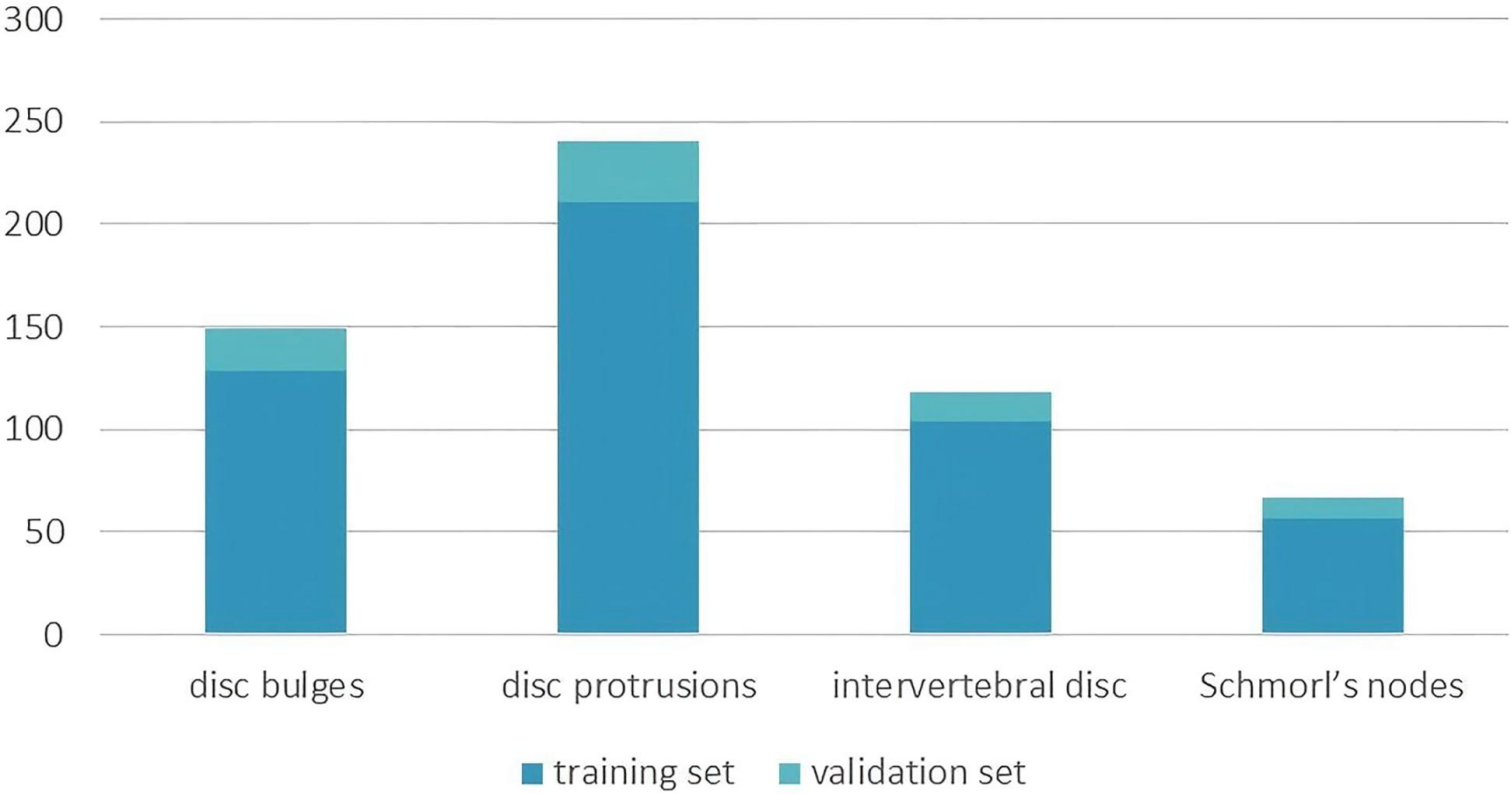
The instance counts for each disc condition in the training and validation sets.

The dataset used in this study was constructed through collaboration with experienced clinicians at our hospital following data preprocessing procedures. The annotation process was performed manually using LabelMe (22), a tool available in Anaconda, to annotate the images. Two board-certified radiologists conducted fully manual contouring for each disc abnormality at the pixel level. The labels were derived solely from the imaging features, adhering to standardized radiological criteria. These annotations were then processed and used for deep learning model training. Using the Imaging Labeling system, radiologists classified and labeled CT images, which were subsequently divided into training and validation sets for deep learning network training. The dataset consists of 574 images in total, with 500 images allocated for training and 74 images for validation. The training set includes images categorized into four intervertebral disc condition types: 104 intervertebral discs, 57 Schmorl's nodes, 128 disc bulges, and 211 disc protrusions. The radiological definitions for these conditions are as follows: normal intervertebral discs are located between two adjacent vertebrae without extending beyond their edges; Schmorl's nodes refer to the protrusion of the nucleus pulposus into the vertebral body through cracks in the upper and lower cartilage end plates; disc bulges are characterized by a protrusion that exceeds 25% of the disc edge or an angle between the two sides of the protrusion and the center of the nucleus pulposus greater than 90°; and disc protrusions are defined by a protrusion of less than 25% of the disc edge, with the base of the protrusion larger than the protrusion itself and an angle between the protrusion sides and the center of the nucleus pulposus of less than 90°. The validation set comprises 14 normal intervertebral discs, 10 Schmorl's nodes, 21 disc bulges, and 29 disc protrusions, and was used to evaluate the model's performance. Figure 1 shows the count of instances for each intervertebral disc condition type across the training and validation sets.

#### 2.2.3 Applying convolutional neural networks to extract features

**Figure 2:**
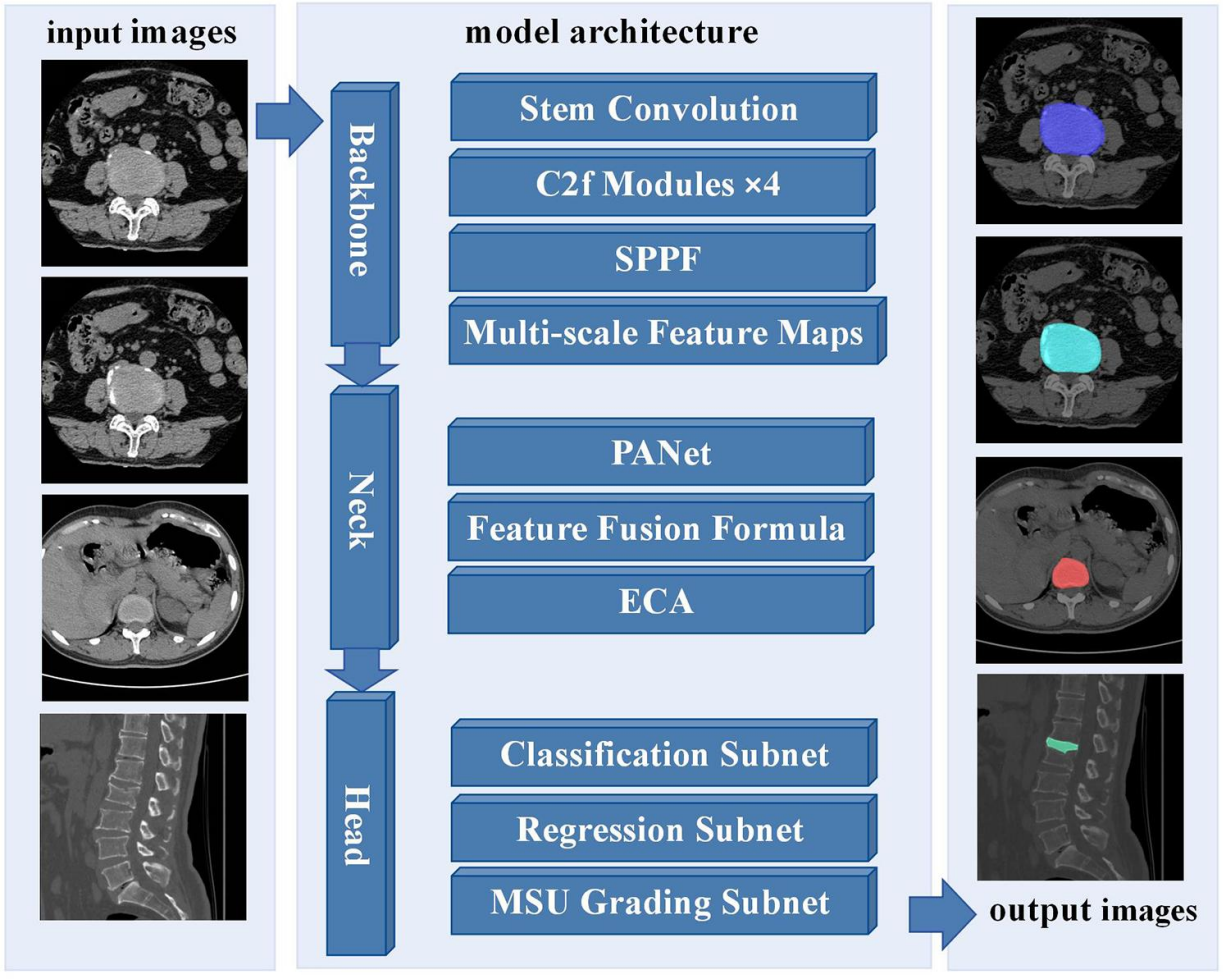
Framework of the proposed YOLOv8-seg-based method for intervertebral disc condition classification.

For the intervertebral disc condition classification task, training images are first input into the YOLOv8-seg network, which is designed to perform both object detection and instance segmentation (23). This end-to-end architecture simultaneously conducts object localization, classification, and pixel-level mask generation. The backbone network extracts multi-scale features, which are integrated through a hybrid FPN-PANet neck to capture detailed disc morphology across varying spatial scales. Figure 2 illustrates the overall framework of the proposed YOLOv8-seg–based method, including input images, model architecture, and output results. In a single forward pass, the network directly outputs bounding boxes, classification scores, and instance masks. A composite loss function is applied, comprising Focal Loss for classification to address sample imbalance, CIoU Loss for bounding box regression to account for overlap, center distance, and aspect ratio, and a Dice–BCE combination for segmentation to optimize both mask boundary delineation and pixel-level accuracy.

#### 2.2.4 Training configuration

The YOLOv8-seg model was trained for 150 epochs with a batch size of 16. An initial learning rate of 0.001 was used for all experiments. These hyperparameters were selected based on empirical performance and training stability observed in preliminary experiments.

### 2.3 Evaluation indicators

In this study, the performance of the automated classification system was evaluated using several key metrics derived from the classification results of the test set. The primary indicators for assessing model performance were the overall correct classification rate and the kappa coefficient. The correct classification rate represents the proportion of correctly classified samples relative to the total number of samples, serving as a general measure of accuracy. The kappa coefficient quantifies the consistency between the model's predictions and the true labels, with values closer to 1 indicating stronger agreement. Given that the YOLOv8-seg model is a multi-class classification network, the evaluation was conducted for each intervertebral disc condition by treating the problem as a multi-class classification task. For each category, the following metrics were computed: sensitivity, specificity, positive predictive value (PPV), negative predictive value (NPV), and the Youden index. Sensitivity (or recall) assesses the model's ability to correctly identify true positives, while specificity evaluates its ability to correctly identify true negatives. PPV reflects the precision of positive classifications, and NPV gauges the reliability of negative classifications. The Youden index combines sensitivity and specificity into a single value, providing a balanced measure of performance across both positive and negative classifications. Collectively, these evaluation metrics provide a comprehensive assessment of the model's ability to accurately classify intervertebral disc abnormalities, ensuring its potential clinical utility and applicability in real-world settings.

## 3 Results

**Figure 3:**
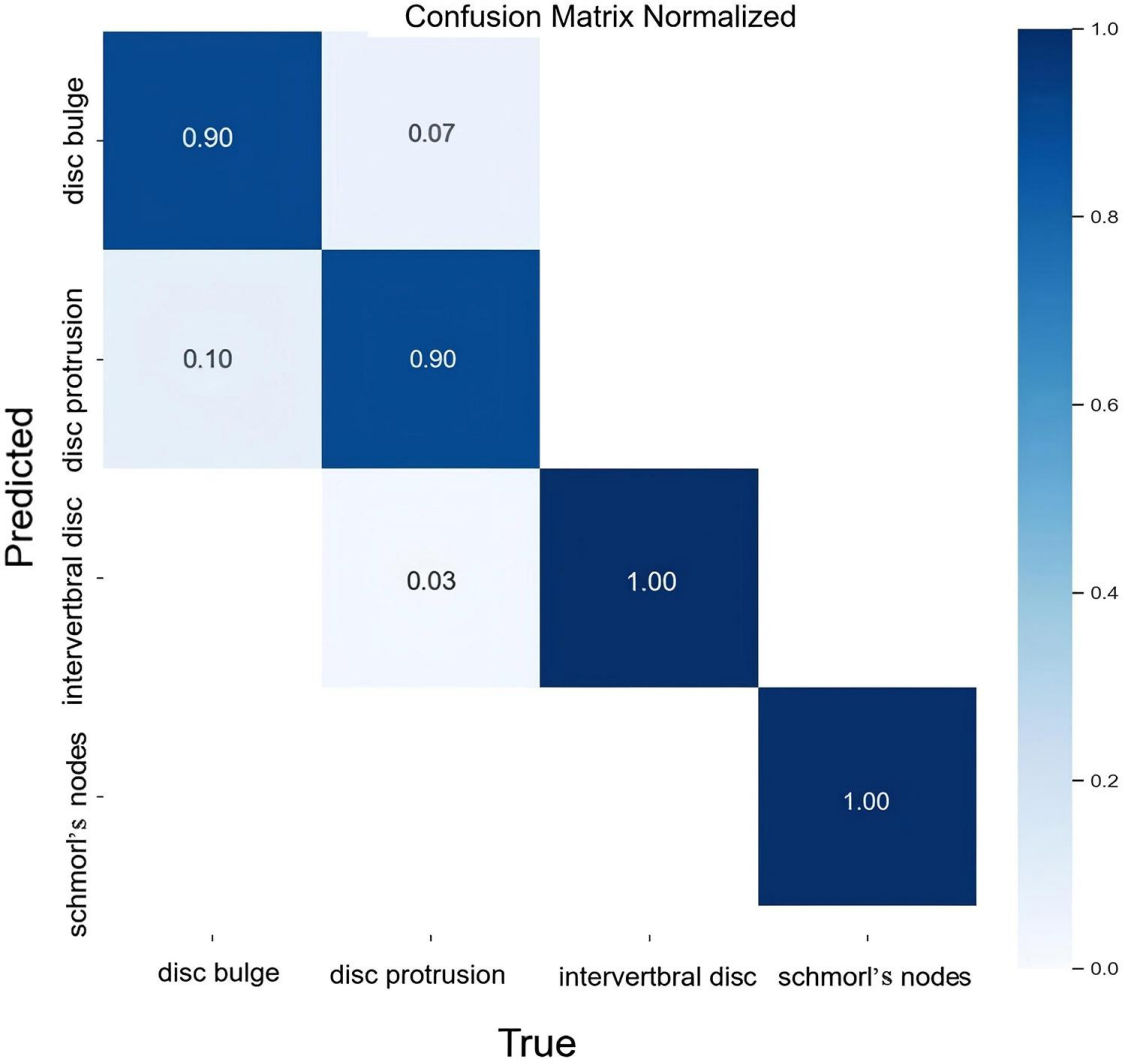
Normalized confusion matrix for intervertebral disc condition classification.

**Figure 4:**
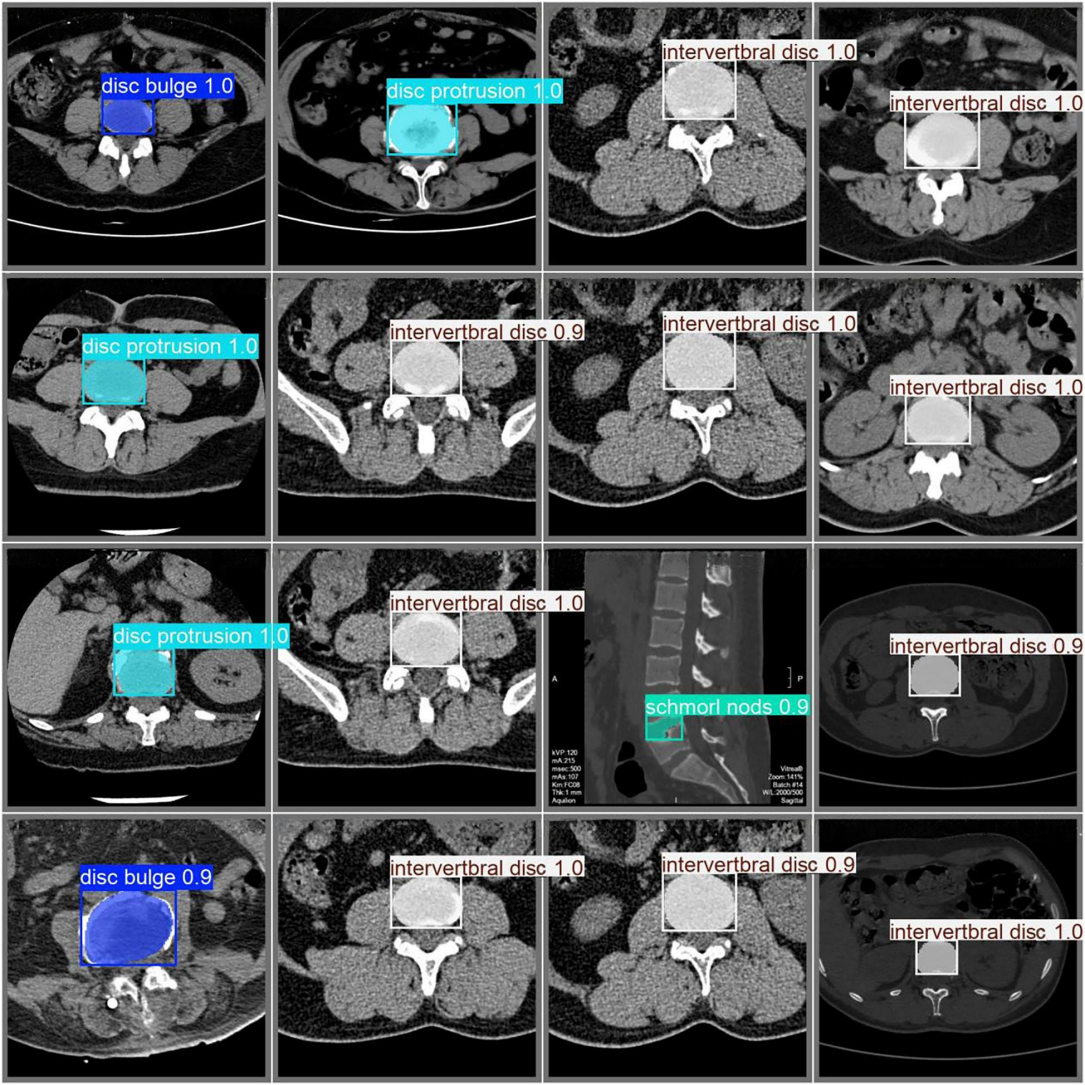
Examples of successful identifications.

**Figure 5:**
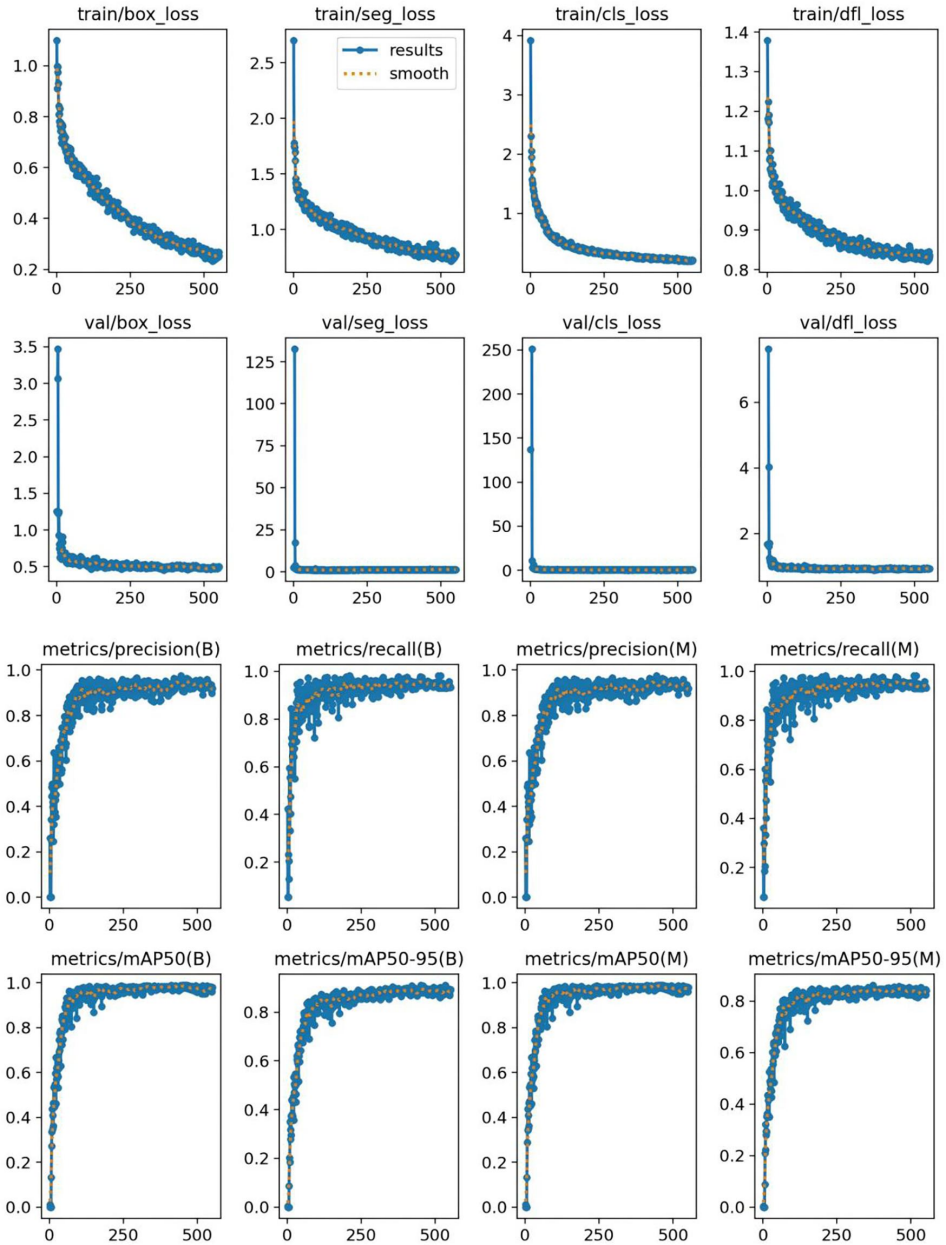
Change curves of loss functions and evaluation metrics during training and validation.

The classification results for intervertebral disc conditions demonstrate the model's high accuracy across multiple categories. Relevant data are provided in Table 1, which details the counts of disc bulge, disc protrusion, intervertebral disc, and Schmorl's nodes in both the true and predicted categories. In the overall classification, comprising 74 images and 74 instances, the model correctly identified 69 instances and misclassified 5. For the “disc bulge” category, which includes 21 samples, 19 were correctly predicted, and 2 were misclassified as disc protrusion. In the “disc protrusion” category, with 29 samples, 26 were correctly identified, while 3 were misclassified as disc bulge. The “intervertebral disc” category, containing 14 samples, was perfectly classified, with all 14 instances correctly identified. Similarly, in the “Schmorl's nodes” category, which included 10 samples, all 10 instances were accurately identified. Figure 3 further emphasizes the model's performance by illustrating the proportions of correct and incorrect predictions for each class. The overall classification performance is further assessed through the model's sensitivity, specificity, positive predictive value, negative predictive value, and Youden index for each category. These metrics provide a thorough understanding of the model's diagnostic efficacy. The corresponding data for these performance metrics are summarized in Table 2. Examples of successful identifications are presented in Figure 4. Figure 5 displays the change curves of loss functions and evaluation metrics throughout the training and validation processes. These curves demonstrate a steady reduction in box loss, segmentation loss, classification loss, and distribution focal loss in both training and validation stages, suggesting the model's improving performance across iterations. Furthermore, evaluation metrics—such as precision, recall, and mAP50—show a continuous increase and stabilization, indicating the model's convergence and enhanced diagnostic accuracy.

**Table 1:** Counts of four categories in true and predicted classes.

True\predicted	Disc bulge	Disc protrusion	Intervertebral disc	Schmorl's nodes
Disc bulge	19	2	0	0
Disc protrusion	2	26	1	0
Intervertebral disc	0	0	14	0
Schmorl's nodes	0	0	0	10

**Table 2:** Data for performance metrics.

Class	Sensitivity	Specificity	Positive predictive value	Negative predictive value	Youden index
Disc bulge	0.905	0.962	0.905	0.962	0.867
Disc protrusion	0.897	0.956	0.929	0.935	0.853
Intervertebral disc	1.000	0.983	0.933	1.000	0.983
Schmorl's nodes	1.000	1.000	1.000	1.000	1.000

## 4 Discussion

Intervertebral disc disease, particularly abnormalities such as bulging and herniated discs and Schmorl's nodes, usually manifests itself clinically as varying degrees of back pain, lumbago, and neurologic dysfunction. However, morphological changes in intervertebral discs are often subtle and these changes manifest differently between patients. Traditional imaging analysis methods rely on physicians’ experience and subjective judgment, which are susceptible to operational errors and differences in diagnosticians’ experience. Therefore, intelligent classification of intervertebral disc abnormalities using automated deep learning methods can not only improve diagnostic accuracy, but also reduce the interference of human factors and significantly improve diagnostic and treatment efficiency (24, 25).

In this study, deep learning techniques were applied to segmentation of intervertebral disc conditions (26). In recent meta-analyses, deep learning-based segmentation models for lumbar intervertebral discs (IVDs) have demonstrated exceptional accuracy, with pooled Dice Similarity Coefficients (DSC) reaching 0.900 (95% CI: 0.887–0.914). Notably, Deeplab variants have achieved DSC values as high as 0.930 in multi-center datasets (27), while U-Net variants have consistently performed with DSC values above 0.897 (28). These findings reinforce the high reliability of deep learning models in IVD segmentation and provide a solid foundation for the anomaly classification task. Large datasets of CT images were screened, preprocessed, renamed, and annotated.

The deep learning-based method for classifying intervertebral disc abnormalities proposed in this study demonstrated significant advantages in automated diagnosis of intervertebral disc disease. To further validate the effectiveness of the proposed YOLOv8-based classifier, we conducted a comprehensive comparison with several baseline models, including YOLOv5, YOLOv3, and Faster R-CNN, using the same dataset. As summarized in Table 3, YOLOv8 consistently outperformed these models across multiple evaluation metrics, achieving an accuracy of 93.2%, compared to approximately 86.5% for YOLOv5, 78.4% for YOLOv3, and 91.9% for Faster R-CNN under identical experimental conditions. These results corroborate previous studies highlighting YOLOv8's enhanced architecture, which delivers superior mean average precision (mAP), particularly in detecting smaller lesions (23). Furthermore, YOLOv8 exhibited significantly lower inference latency than Faster R-CNN, enhancing its suitability for real-time clinical applications (29, 30). Similar comparative studies report YOLOv8 mAP50 values ranging from 71% to 94%, consistently surpassing those of Faster R-CNN (31). Collectively, these findings confirm that YOLOv8 provides an optimal b alance of accuracy, sensitivity, and speed, reinforcing its suitability for accurate and efficient intervertebral disc classification.

**Table 3:** Comparison of classification results on the same dataset.

Model	Correct/Total	Accuracy (%)
YOLOv8	69/74	93.2%
YOLOv5	64/74	86.5%
YOLOv3	58/74	78.4%
Faster R-CNN	68/74	91.9%

By using the YOLOv8-seg model to learn from preprocessed data, a segmentation model is built and disc conditions are segmented. We not only achieved a 93.2% correct classification rate in terms of accuracy, but also obtained a kappa coefficient of 0.905 (*P* < 0.001), indicating that the model has a high degree of reliability and consistency in clinical practice. The classification model demonstrated strong performance in four categories of intervertebral disc disease. Both the “intervertebral disc” and “Schmorl's node” categories achieved perfect sensitivity and specificity values of 1.00, reflecting the clear and easily distinguishable characteristics of these diseases on medical imaging. In contrast, the sensitivities for “bulging disc” and “herniated disc” were slightly reduced (0.905 and 0.897, respectively), although their specificities remained high (0.962 and 0.956), indicating the model's effectiveness in accurately excluding these conditions. The reduced sensitivity may be due to the morphological similarity between bulging and protruding discs, which may lead to occasional misclassification. Nevertheless, the model demonstrated strong positive predictive value (PPV) and negative predictive value (NPV) in all categories, with “Schmorl's Nodes” achieving a perfect PPV and NPV score of 1.00. The overall performance of the model is further emphasized by the Youden Index, with Schmorl's Nodes achieving the highest value (1.00), followed by Disc Bulge at 0.983. While Disc Bulge and Disc Protrusion achieve a perfect PPV and NPV score of 1.00, Schmorl's Nodes achieves a perfect PPV and NPV score of 1.00. “Disc Bulge” and “Disc Protrusion” were slightly lower, the model maintained a good balance between sensitivity and specificity, especially in minimizing false positives. The above results indicate that the model effectively differentiates between various disc diseases. And compared to previous studies, we accurately differentiated between pathologic patterns such as bulging and herniated discs with greater accuracy (26, 32). It demonstrates the strong potential of the model in the diagnosis of intervertebral disc diseases, which can effectively compensate for the limitations in the traditional manual image analysis methods.

Combined with deep learning technology, the automated classification of intervertebral disc abnormalities not only provides strong support in imaging screening, but also facilitates early diagnosis and follow-up management of the disease (33). In the early stages of degenerative disc disease, automated identification using AI models can help detect signs of disease early, leading to individualized intervention and treatment. In addition, during post-operative follow-up, automated analysis based on deep learning can effectively monitor the disc health status of post-operative patients, preventing and timely detecting potential complications, such as the progression of degenerative disc changes or post-operative complications. With the automated analysis of the AI system, the patient's diagnosis will be more accurate, which is conducive to the clinician's adoption of an individualized treatment plan, thus optimizing the allocation of medical resources.

Despite the relatively favorable results of this study, the clinical application of deep learning models still faces some challenges. In clinical practice, image features of disc abnormalities have large individual variations, and different scanning equipment and scanning parameters may affect image quality and feature extraction. A key limitation of this study is that the dataset is relatively small, containing 574 images, which may not fully capture the range of variations encountered in clinical practice. This constraint may undermine the model's ability to generalize, as deep learning models typically require large, diverse datasets to effectively distinguish subtle differences between conditions. In addition, class imbalances in the dataset may affect model performance. Specifically, a dataset containing a disproportionate number of herniated disc cases may bias the model toward better performance in that class. In contrast, cases with smaller samples, such as Schmorl nodes, may be underrepresented, which reduces the model's sensitivity to these less common cases. Therefore, in future studies, the training dataset needs to be further extended to cover more multicenter data to improve the model's adaptability to different types and devices. While the conditions of normal, bulge, and protrusion discs can be clearly distinguished according to established diagnostic guidelines, we fully acknowledge that a disc may present both protrusion and Schmorl's node. In our current study, we addressed only a single condition at a time. This limitation will be addressed in future research, where we plan to explore multi-label cases, enabling the model to better handle such complex scenarios and enhance its diagnostic accuracy for combined pathologies.

## 5 Conclusion

In summary, this study tackles the challenges associated with diagnosing intervertebral disc abnormalities using traditional methods and proposes a deep learning-based classification approach for automated diagnosis. The YOLOv8-seg model presented in this research effectively classifies four types of intervertebral disc conditions—intervertebral disc, Schmorl's nodes, disc bulges, and disc protrusions—thereby aiding in the diagnostic process, alleviating the workload of radiologists, and enhancing diagnostic consistency. The model's strong performance, particularly in terms of specificity, positive predictive value, and negative predictive value, underscores its potential to improve clinical decision-making and patient outcomes.

Nevertheless, the study has certain limitations. The relatively small sample size used for training and validation may hinder the model's generalization to larger and more diverse populations, and some misclassifications, especially between disc bulges and disc protrusions, persist. Future research will aim to overcome these limitations by expanding the dataset and exploring alternative model architectures and techniques to further enhance classification accuracy and robustness in varied clinical environments. Furthermore, cross-validation will be incorporated in future work to ensure more reliable and unbiased performance evaluation. Multicenter validation is also necessary to assess the model's effectiveness across different imaging protocols, scanner types, and patient populations. Such validation would enhance the reliability of the findings and help uncover potential biases associated with single-center data. In this study, we focused on single-condition cases. This limitation will be addressed in future research, where we plan to explore multi-label scenarios and expand our analysis to enhance the model's ability to classify more complex, coexisting pathologies.

## Data Availability

The raw data supporting the conclusions of this article will be made available by the authors, without undue reservation.
